# The effects of theaflavin-enriched black tea extract on muscle soreness, oxidative stress, inflammation, and endocrine responses to acute anaerobic interval training: a randomized, double-blind, crossover study

**DOI:** 10.1186/1550-2783-7-11

**Published:** 2010-02-23

**Authors:** Shawn M Arent, Meghan Senso, Devon L Golem, Kenneth H McKeever

**Affiliations:** 1 Department of Exercise Science & Sport Studies, Rutgers University, New Brunswick, NJ, USA; 2 Department of Nutritional Sciences, Rutgers University, New Brunswick, NJ, USA; 3 Department of Endocrinology & Animal Biosciences, Rutgers University, New Brunswick, NJ, USA

## Abstract

**Background:**

Muscle soreness and decreased performance often follow a bout of high-intensity exercise. By reducing these effects, an athlete can train more frequently and increase long-term performance. The purpose of this study is to examine whether a high-potency, black tea extract (BTE) alters the delayed onset muscle soreness (DOMS), oxidative stress, inflammation, and cortisol (CORT) responses to high-intensity anaerobic exercise.

**Methods:**

College-age males (N = 18) with 1+ yrs of weight training experience completed a double-blind, placebo-controlled, crossover study. Subjects consumed the BTE (1,760 mg BTE·d^-1^) or placebo (PLA) for 9 days. Each subject completed two testing sessions (T1 & T2), which occurred on day 7 of the intervention. T1 & T2 consisted of a 30 s Wingate Test plus eight 10 s intervals. Blood samples were obtained before, 0, 30 & 60 min following the interval sessions and were used to analyze the total to oxidized glutathione ratio (GSH:GSSG), 8-isoprostane (8-iso), CORT, and interleukin 6 (IL-6) secretion. DOMS was recorded at 24 & 48 h post-test using a visual analog scale while BTE or PLA continued to be administered. Significance was set at *P < 0.05*.

**Results:**

Compared to PLA, BTE produced significantly higher average peak power (*P = 0.013*) and higher average mean power (*P = 0.067*) across nine WAnT intervals. BTE produced significantly lower DOMS compared to PLA at 24 h post test (*P < 0.001*) and 48 h post test (*P < 0.001*). Compared to PLA, BTE had a slightly higher GSH:GSSG ratio at baseline which became significantly higher at 30 and 60 min post test (*P < 0.002*). AUC analysis revealed BTE to elicit significantly lower GSSG secretion (*P = 0.009*), significantly higher GSH:GSSG ratio (*P = 0.001*), and lower CORT secretion (*P = 0.078*) than PLA. AUC analysis did not reveal a significant difference in total IL-6 response (*P = 0.145*) between conditions.

**Conclusions:**

Consumption of theaflavin-enriched black tea extract led to improved recovery and a reduction in oxidative stress and DOMS responses to acute anaerobic intervals. An improved rate of recovery can benefit all individuals engaging in high intensity, anaerobic exercise as it facilitates increased frequency of exercise.

## Background

Recovery after high intensity exercise is becoming increasingly important as sport and exercise become more competitive. After a high-intensity bout of exercise, muscle soreness, decreased power, and decreased performance often follow [1-3]. By reducing the magnitude and length of these effects, an athlete may be able to train more frequently and increase long-term performance. Antioxidant and anti-inflammatory supplements, such as theaflavins found in black tea, have been suggested to decrease oxidative stress and inflammation resulting from physiological stressors [4-8]. This leaves reason to investigate whether a supplement such as a high-potency black tea extract (BTE) could positively impact delayed-onset muscle soreness (DOMS) and the precipitating biochemical and hormonal responses.

DOMS typically occurs after unaccustomed or high-intensity exercise, most commonly anaerobic [1-3]. Soreness is usually noted at 24 hours post-exercise and can last as long as 5 to 7 days post-exercise [1]. Although several models of DOMS have been suggested, researchers generally agree that muscle damage initiates a cascade of events leading to DOMS [1,3,9-11]. The muscle damage and oxidative stress response following anaerobic exercise have been deemed necessary to promote skeletal muscle remodeling [1,10-13] to gain benefit from the exercise, but enhanced recovery may be advantageous for more rapidly promoting an anabolic environment.

Exercise elicits mechanical and hormonal reactions from the body. The resulting muscle damage from these reactions elicits inflammatory and oxidative responses that may exacerbate muscle injury and prolong the time to regeneration [1,3,11]. The hormonal contributor to muscle damage during exercise is derived through basic neuroendocrine responses to exercise demands. High intensity exercise triggers the activation of the hypothalamic-pituitary-adrenal (HPA) axis leading to the release of cortisol and other catabolic hormones. These hormones function to meet increased energy needs by recruiting substrates for gluconeogenesis via the breakdown of lipids and proteins. Through their catabolic nature, these hormones also indirectly lead to muscle cell damage [12].

Inflammation following anaerobic exercise functions to clear debris in preparation for muscle regeneration [1,9]. The magnitude of the increase in inflammatory cytokines (such as IL-6) varies proportionately to the intensity and duration of the exercise [14,15]. However, a prolonged inflammatory response can increase muscle damage and delay recovery by exacerbating oxidative stress and increasing production of reactive oxygen species (ROS) [16]. The increased ROS production seen with high intensity training [12,17] can lead to oxidative stress such as lipid peroxidation [1,18].

Theaflavins, which are commonly found in black tea, have been suggested to reduce oxidative stress [6-8] by acting as an antioxidant with radical-scavenging ability [4]. Furthermore, the theaflavin-enriched black tea extract (BTE) used in this study has been previously shown to reduce inflammation and the production of inflammatory cytokines, including IL-6, in the mouse model [19]. However, most of the antioxidant and anti-inflammatory effects of theaflavins have been examined with regards to disease. There is little information regarding theaflavins' effect on inflammation, oxidative stress, and related systemic responses to exercise or on the exercise-induced DOMS model in humans. Antioxidant supplementation may help buffer the excessive stress of high intensity exercise or potentially enhance recovery, which ultimately may result in a reduction in DOMS.

The purpose of this study was to examine the impact of supplementing with a theaflavin-enriched black tea extract (BTE) on delayed onset muscle soreness (DOMS), oxidative stress, cortisol, and inflammatory responses to a high-intensity anaerobic exercise protocol. Given the interrelated nature of HPA axis activation, inflammatory cytokine production, and formation of reactive oxygen species (ROS), it was hypothesized that BTE would improve recovery from an acute bout of intense exercise. Additionally, it was predicted that the enhanced recovery and reduced inflammation would positively influence the ratings of DOMS at 24 and 48 hours post-exercise.

## Methods

### Subjects

A total of 18 college-age males (M_age _= 21.3 ± 0.4 yrs; M_weight _= 84.3 ± 2.5 kg; M_height _= 175.8 ± 2.0 cm) with 1+ years of weight training experience (M_experience _= 5.4 ± 0.7 yrs) (at least 3 days per week) completed all stages of data collection. Initially, 24 subjects enrolled in the study. However, one subject dropped out due to training conflicts with his sport. The other 5 subjects withdrew of their own volition due to an inability to tolerate the physical demands of the testing protocol. This study was limited to males in order to control for fluctuations in cortisol that occur during the menstrual cycle. Risks and benefits were explained to the subjects and each of them gave written informed consent prior to participation in the study. At initial enrollment, all subjects self-reported to be free from current injuries limiting their ability to train and complete physiological testing. Additionally, all subjects were asked to refrain from using anti-inflammatory medication or drinking tea during the course of the study. Subjects were asked about supplement use within the past 6 months. Those subjects on supplements were directed to continue to use the supplement at the current dosage throughout the entire study provided they had been consuming the supplement for at least one month prior. If they had not been using the supplement for at least one month, they discontinued the use of the supplement and completed a 2-week washout phase prior to commencing with the study. Each subject was screened by a member of the research team prior to commencing with each day of testing in order to assess compliance to supplementation and adherence to the exclusion criteria. Prior to enrollment in the study, a health screening was also completed with each subject in accordance with American College of Sports Medicine (ACSM) exercise testing procedures.

### Study Design and Supplementation

A double-blind, crossover design was used for this study. Each subject completed a familiarization session to control for practice effects on the anaerobic test [20] and two separate testing sessions (T1 and T2). During T1 and T2, participants had body composition assessed and blood samples were obtained before, immediately after, 30- and 60-min after a Wingate Anaerobic Test (WAnT) for later analysis of oxidative stress markers (8-iso PGF_2α _[8-isoprostane], total and oxidized glutathione [GSH and GSSG]), cortisol (CORT), and inflammatory cytokine (interleukin 6 [IL-6]). Additionally, capillary blood samples were analyzed during each test in order to assess blood lactate accumulation and recovery. Participants were asked to rate perceived muscle soreness at 24 and 48 h post on a visual analog scale. Subjects were required to refrain from training for 24 h prior to each test and to refrain from lower body training for at least 24 h post. Additionally, each subject was tested at the same time of day for each test to control for diurnal variations. Participants were instructed to continue with their normal exercise training during the study.

Following the familiarization session, which included health screening and an initial WAnT plus one interval, the subjects were randomly assigned to order of administration of the theaflavin-enriched BTE (WG0401; WellGen, New Brunswick, NJ) and a placebo (PLA). The bottles of the BTE and PLA were provided in a blinded fashion by WellGen, Inc. with a de-coding list secured from the investigators until the completion of all assays. Un-blinding occurred at the completion of data processing in order to facilitate data entry. All subjects acknowledged receipt of each bottle and the bottles were returned following each phase of the study to allow for a count of the remaining capsules. Based on the return, 100% compliance was achieved.

The BTE used in the study contains at least 40% theaflavins, including theaflavin (TF), theaflavin-3-gallate (TF-3-G), theaflavin-3'-gallate (TF-3'-G), and theaflavin-3,3'-digallate (TF-3,3'-diG). It also contains approximately 30% catechins and total polyphenols exceeding 95%. Subjects were instructed to take two capsules in the morning and two in the early afternoon. Each 2-capsule serving of the experimental product contains ~880 mg BTE and is standardized for 350 mg TF. Placebo was matched for appearance.

The initial supplement phase commenced 2 to 3 days following the familiarization session in order to allow residual muscle soreness and muscle damage to subside. Subjects consumed the BTE or PLA for 9 days. T1 occurred on day 7 and administration continued for 2 more days during the assessment of DOMS. Each subject then underwent a 5-day washout before beginning the 9-day administration period of whichever product they did not receive in the initial supplementation phase. As with the first phase, T2 occurred on day 7 of the second phase and administration continued for an additional 2 days during the assessment of DOMS. The timeline was as follows: Day 1, familiarization; Days 3-11, supplement phase 1 (testing on day 9); Days 12-16, washout phase; Days 17-25, supplement phase 2 (testing on day 23). Subjects were directed to maintain their usual diet and avoid drastic changes in consumption. A 3-day dietary recall log was used for each subject prior to each trial and analyzed using commercially available dietary analysis software (FoodWorks, Xyris Software) to assess dietary changes from T1 to T2. Analyses indicated no differences in dietary intake (*P > 0.34)*.

### Exercise Test Procedures

For each testing day, all subjects reported to the Rutgers University Human Performance Laboratory. Subjects were asked to arrive normally hydrated, to have eaten a high carbohydrate meal 2 h prior, and to refrain from ingesting substances that could affect normal physiological functioning (i.e., tea, coffee, alcohol, nicotine). Satisfaction of these criteria was confirmed prior to commencing with testing. Following this, each subject rested in a supine position for 10 min before commencing with the pretest blood draw. Blood samples were also obtained immediately following completion of the exercise test and at 30 and 60 min post-test with the subject in a supine position.

Subjects performed the WAnT during each testing day on a Monark 894E Anaerobic Test Ergometer (Monark Exercise AB, Sweden). The load was set according to each subject's mass [21]. The test was a 30-second WAnT followed by 5 min of rest and then eight 10-s intervals of all-out cycling. Each interval was separated by 2 min of rest. The resistance for the WAnT and intervals was set at 0.10 kP/kg body mass.

### Performance Measures

Peak power during the WAnT was defined as the highest mechanical power output elicited during each 30 s test. Mean power was calculated based on the average mechanical power produced during the test. Additionally, average peak power output and average mean power output were both calculated across the WAnT and all 8 intervals.

### Biochemical Measures

Capillary blood samples (5 μL) were taken from the fingertip during the baseline resting blood draw and at 0, 5, and 10 min post-exercise in order to determine peak blood lactate values and clearance. The Lactate Pro (Arkray, Japan) portable analyzer was used to determine whole blood lactate content.

Before (t0), immediately after (t1), 30 min post (t2), and 60 min post (t3) each WAnT + interval session, blood samples were collected via an indwelling cannula inserted into an antecubital vein using a vacutainer system (Becton Dickinson, Rutherford, NJ). Approximately 10 mL were collected in a serum separator tube and 10 mL in an EDTA coated tube. After removing a 1 mL aliquot of whole blood for hemoglobin and hematocrit analysis to account for plasma volume changes, an additional 300 μL aliquot (2 × 100 μL for GSSG; 2 × 50 μL for GSH) was obtained for GSH/GSSG analysis. 1-methyl-2-vinylpyridium (M2VP) was added to the tubes containing samples for GSSG analysis. Plasma for 8-isoprostane assay was obtained by centrifugation of whole blood in the EDTA tubes at 3000 × g 10 min at 4°C with 1 mL aliquots placed in microvials pre-coated with 200-μg of butylatedhydroxytoluene (BHT). The serum separator tubes were left to stand for 30 min to facilitate clotting before being centrifuged at 3500 × g for 15 min at 4°C in order to obtain serum for IL-6 and CORT analysis. Aliquots of blood, serum, and plasma were stored at -80°C until analysis of the dependent measures. All assays were performed in duplicate and assays for each measure were run in one batch.

Total and oxidized glutathione were analyzed using a commercially-available EIA kit (Bioxytech^® ^GSH/GSSG-412, OxisResearch, Portland, OR). The within assay coefficient of variation (CV) for GSH was ± 7.3% and for GSSG was ± 8.6%. Similarly, IL-6 was determined via ELISA using commercial kits (IBL, Hamburg, Germany). Within assay CV for IL-6 was ± 6.9%. Serum CORT was analyzed using RIA (MP Biomedicals, Irvine, CA), and the within assay CV was ± 6.2%.

In order to analyze plasma free 8-iso PGF_2α_, plasma from the EDTA tubes was first purified by diluting the sample in a 1:5 ratio with Eicosanoid Affinity Column Buffer (Cayman Chemical, Ann Arbor, MI). A known amount of tritiated 8-iso PGF_2α _was added prior to purification in order to determine recovery rates. Ethanol was added to the solution and the sample was chilled at 4°C for 5 min to precipitate proteins, and then centrifuged at 1500 × g for 10 min at 4°C. The supernatant was decanted and the remaining ethanol evaporated under a nitrogen stream. The pH was then lowered to 4.0 using dropwise addition of HCl. Samples were then passed through a C-18 affinity column (Cayman Chemical, Ann Arbor, MI) previously activated with methanol and UltraPure water. Following addition of the sample, the column was washed with 5 mL UltraPure water followed by 5 mL HPLC grade hexane (Sigma Chemical, St. Louis, MO). The sample was then eluted with 5 mL of an ethyl acetate:methanol solution (Cayman Chemical, Ann Arbor, MI). The elution solution solvents were evaporated again under nitrogen and the samples were then reconstituted in 450 μL EIA buffer (Cayman Chemical, Ann Arbor, MI). For each purified sample, 50 μL was analyzed using a commercially available 8-isoprostane EIA kit (Cayman Chemical, Ann Arbor, MI), with each sample assayed in duplicate. Absorbance values were determined with a Spectramax 340 microplate reader (Molecular Devices, Sunnyvale, CA) between 405 nm and 420 nm and the raw data corrected using the recovery rates of tritiated PGF_2α _. The within assay CV for 8-iso was ± 8.7%

### Delayed Onset Muscle Soreness

A 10 cm visual analog scale (VAS) was used to determine perceived muscle soreness. The anchors at 0 and 10 cm corresponded to "no soreness" and "too sore to move muscles", respectively. Subjects were asked to perform one squat with hands on hips and then draw a line on the scale corresponding to their level of soreness [2]. Subjects completed the assessments at 24 and 48 h post testing at T1 and T2.

### Statistical Analysis

Peak power, average peak power, mean power, and average mean power were analyzed using repeated measures ANOVAs. A series of 2 × 4 (condition × time) repeated measures ANOVAs were used to analyze LAC, CORT, GSH:GSSG, and 8-iso. DOMS responses were analyzed using a 2 × 2 (condition × time) repeated measure ANOVA. For each of the above analyses, simple effects and simple contrasts were used as follow-ups where appropriate. After assessing skewness statistics for the data, log_10 _transformations were used to normalize data for GSSG, GSH:GSSG ratio, 8-iso, CORT, and IL-6. Finally, area under the response curve (AUC) for each biochemical variable was calculated using trapezoidal integration in order to determine total secretion responses. AUC for each variable was then analyzed using individual repeated measure ANOVAs. Skewness was assessed for AUC and log_10 _transformations were again applied to GSH, GSSG, GSH:GSSG ratio, 8-iso, CORT, and IL-6. For each univariate analysis, examination of the Huynh-Feldt (H-F) epsilon for the general model was used to test the assumption of sphericity. If this statistic was greater than 0.75, sphericity was considered to have been met and the unadjusted univariate statistic was used. If epsilon was less than 0.75, a violation of the assumption of sphericity was considered to have occurred and the H-F adjusted statistic was used to determine significance. Significance was set at α < 0.05. Trends were identified and discussed if *P *< 0.10.

## Results

### Performance

**Figure 1:**
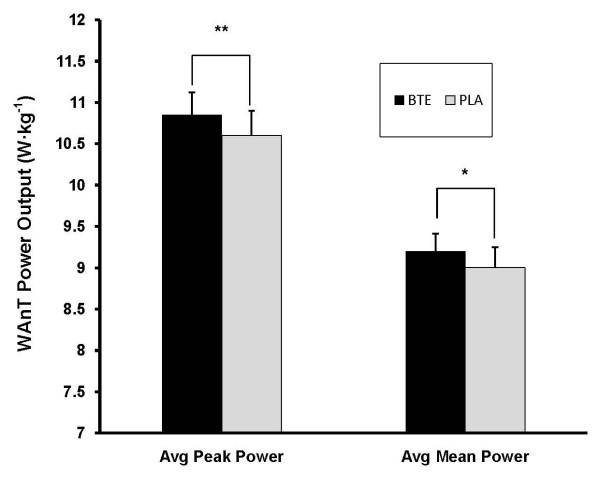
**Average Peak Power and Average Mean Power over nine WAnT intervals for BTE and PLA conditions**. Data (mean ± SE) are expressed as W·kg^-1^. BTE produced significantly higher values than PLA in both parameters. ** represents (P < 0.05) difference between conditions. * represents (P < 0.10) difference between conditions.

No significant differences were observed between BTE and PLA on either peak power (*P = 0.111*) or mean power (*P = 0.395*) during the 30 s WAnT. However, when peak power and mean power were averaged across the entire session consisting of the 30 s WAnT and eight 10 s intervals, differences between conditions did emerge. Compared to PLA, BTE produced significantly higher average peak power (BTE = 10.85 ± 0.27 W·kg^-1^; PLA = 10.6 ± 0.30 W·kg^-1^, *P = 0.013*) and a trend for higher average mean power (BTE = 9.2 ± 0.21 W·kg^-1^; PLA = 9.0 ± 0.25 W·kg^-1^, *P = 0.067*). See Figure 1.

### Delayed Onset Muscle Soreness

**Figure 2:**
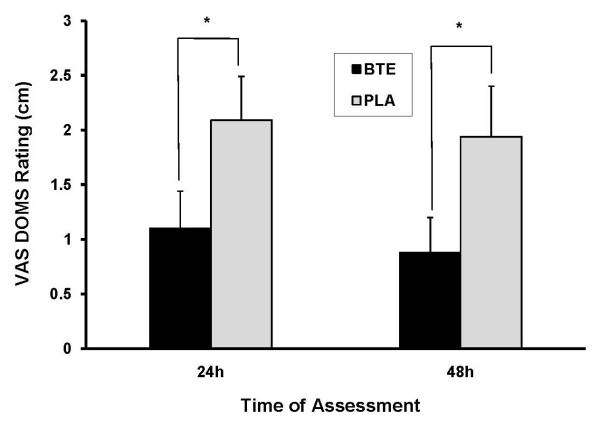
**DOMS Ratings 24 h and 48 h post-exercise in BTE vs PLA conditions**. Data (mean ± SE) are expressed as cm ratings obtained from visual analog scale. Compared to PLA, BTE produced significantly lower DOMS ratings 24 h and 48 h post exercise. * represents (P < 0.001) difference between conditions.

A significant condition main effect emerged for DOMS (*P < 0.001*). Across the 48 h post-exercise period, BTE produced significantly lower DOMS ratings (24 h = 1.12 ± 0.34 cm; 48 h = 0.88 ± 0.32 cm) compared to PLA (24 h = 2.09 ± 0.40 cm; 48 h = 1.94 ± 0.46 cm). See Figure 2.

### Biochemical & Hormonal Responses

#### Lactate

A significant time (*P < 0.001*) main effect emerged for lactate. Compared to baseline values, both BTE and PLA had significant elevations in LAC at 0, 5, and 10 min post-exercise (*P < 0.001*). A trend for a condition effect (*P = 0.092) *appeared to emerge due to slightly higher LAC concentrations in the BTE condition at 0 min post-exercise (*P = 0.034*). However, there were no differences in the pattern of LAC response (*P = 0.18*).

#### Oxidative Stress

**Figure 3:**
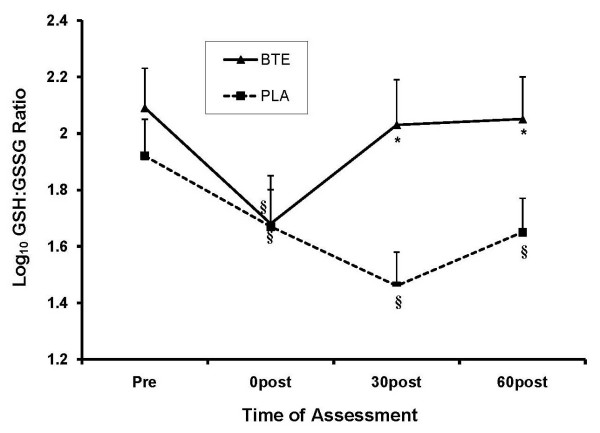
**Effect of BTE vs PLA on plasma GSH:GSSG ratio at baseline, 0, 30, and 60 min post exercise**. Data were normalized via log_10 _transformation. BTE had higher GSH:GSSG ratio at 30 and 60 min post exercise compared to PLA. § represents (P < 0.001) difference from baseline within condition. * represents (P < 0.01) difference between conditions within time.

A significant time main effect (*P = 0.005*) and a trend for a time × condition interaction (*P = 0.056*) emerged for GSH. The interaction appears to be primarily due to slightly higher baseline GSH in the BTE condition, which is an indicator of antioxidant status. There were no differences in GSH AUC (*P = 0.94*). GSSG also demonstrated a significant time main effect (*P < 0.001*), a significant condition main effect (*P = 0.002*) and a significant time × condition interaction (*P < 0.001*). There were equivalent GSSG responses (*P = 0.35) *immediately after exercise (0 post), but the oxidative stress was buffered much more quickly by BTE, with significantly lower GSSG in the BTE condition by 30 min post (*P < 0.001*) and persisting through 60 min post (*P = 0.004*). There was a significant difference in AUC between conditions in favor of BTE (*P = 0.009*). Additionally, a significant condition main effect (*P = 0.004*), a significant time main effect (*P < 0.001*), and a significant time × condition interaction (*P < 0.001*) emerged for the GSH:GSSG ratio. See Figure 3. A lower/decreasing ratio indicates greater oxidative stress as GSSG is prevented from reconverting to GSH. In this case, BTE had lower overall oxidative stress at 30 and 60 min post compared to PLA (*P < 0.002*). The AUC analysis for GSH:GSSG was significant (*P = 0.001*), with an overall greater ratio seen for the BTE condition.

There was a significant time main effect for 8-iso (*P = 0.026*) due to elevated 8-iso secretion following exercise for both conditions. AUC analysis did not reveal significant differences in overall 8-iso secretion (*P = 0.312*).

#### Cortisol

**Figure 4:**
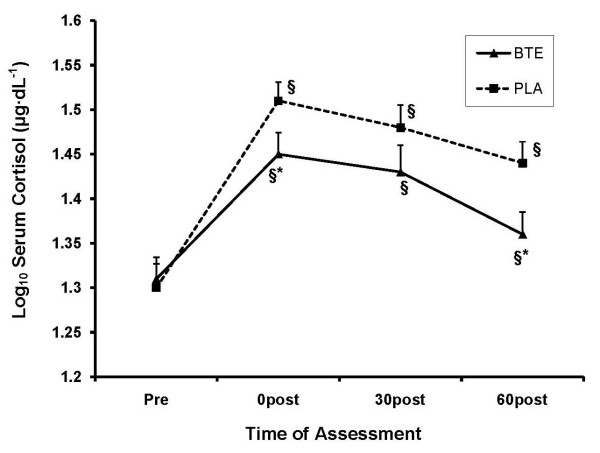
**Effect of BTE vs PLA on cortisol secretion at baseline, 0, 30, and 60 min post exercise**. Data were normalized via log_10 _transformation. BTE produced lower CORT secretion compared to PLA at 0 min and 60 min post exercise. § represents (P < 0.05) difference from baseline within condition. * represents (P < 0.10) difference between conditions within time.

A significant time (*P < 0.001*) main effect and a trend for a condition main effect (*P = 0.078*) emerged for CORT secretion. Though both conditions produced elevated CORT values post-exercise, the BTE condition had lower overall CORT secretion. The time × condition interaction was significant (*P = 0.042*), revealing that HPA recovery is either more pronounced in BTE or that overall HPA activation was not as pronounced. Though all post-exercise assessments revealed higher CORT for both BTE (*P < 0.024*) and PLA (*P < 0.001*) compared to baseline, CORT was lower in BTE compared to PLA immediately post-exercise (*P = 0.074*) and significantly lower at 60 min post-exercise (*P = 0.020*). See Figure 4. Consistent with the interaction, AUC analysis also approached significance (*P = 0.078*), indicating lower total CORT secretion over the duration of recovery with BTE.

#### IL-6

**Figure 5:**
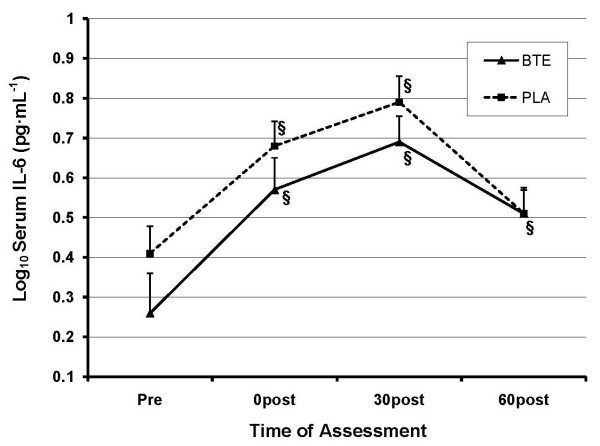
**Effect of BTE vs. PLA on serum IL-6 levels at baseline, 0, 30, and 60 min post exercise**. Data were normalized via log_10 _transformation. A time main effect for IL-6 occurred across both conditions. No significant differences in IL-6 levels between conditions were observed. § represents (P < 0.001) difference from baseline within condition.

A significant time main effect emerged for IL-6 (*P < 0.001*), with a continued rise in IL-6 in both conditions until 30 min post before beginning to return towards baseline. IL-6 production was slightly higher in PLA, though this was not significant (*P = 0.112*). See Figure 5. AUC analysis revealed no significant differences in total IL-6 response between BTE and PLA (*P = 0.145*).

## Discussion

Results of the current study indicate that, in comparison to the placebo, supplementation with BTE resulted in increased baseline antioxidant status, reduced oxidative stress response to anaerobic exercise, improved HPA axis recovery, greater average peak power and average mean power across nine WAnT intervals, and lower DOMS ratings 24 and 48 hours after anaerobic exercise. These findings may hold practical significance for physically active individuals or athletes involved in training that requires high intensity, acute bouts of anaerobic exercise. It is possible that the antioxidant benefits from black tea may translate beyond disease and can have potential for improving recovery of oxidative stress and inflammation after exercise.

The reduced oxidative stress response observed with BTE supplementation may be due to the antioxidant effects of the theaflavin component of the BTE supplement, or to the ability of the BTE supplement to increase endogenous antioxidants such as GSH. Consistent with the oxidative stress results, the high intensity anaerobic exercise with BTE supplementation also elicited a lower cortisol secretion. Possible explanations include blunting of the activation of HPA axis or potentially the recovery from HPA axis activation was more enhanced.

It is important to note that oxidative stress occurred post-exercise under both conditions (supplementation with BTE or PLA). Elimination of this response would be undesirable as it is essential for muscle repair and hypertrophy [1,10,12]. The oxidative marker results reveal that oxidative stress was initiated to similar degrees across conditions yet the recovery was more pronounced with BTE supplementation.

Previous research has revealed that IL-6 is the most responsive cytokine to exercise [22] and its appearance in the blood not only precedes the other cytokines but is also more distinct [15]. In this study, plasma IL-6 significantly increased post-exercise and remained elevated through 60 min of recovery in both BTE and PLA. Though, graphically, the response appeared to be slightly blunted for BTE, this result was not significant. It is likely that the previously demonstrated anti-inflammatory effects of BTE [19] were overpowered by the sheer intensity of the anaerobic exercise testing protocol used in the current study. The magnitude of the CORT and oxidative stress responses serve to reinforce this notion. It is also possible that any anti-inflammatory effects of BTE appeared later in the recovery period after assessments were completed at 60 min post. Given the extended time-course of some cytokines and in light of the DOMS results for the current study, future research is needed to examine the effects of BTE during an extended post-exercise assessment period.

The increase in overall average peak and mean power with BTE supplementation implies increased performance with BTE which may be due to increased recovery between intervals of the WAnT protocol. As well, the blood lactate levels were higher with BTE supplementation at 0 and 5 min post high intensity exercise which is consistent with the higher workload completed. Based on these power and lactate results, the BTE supplementation appears to have resulted in the performance of more total work, which amplifies the significance of the biochemical and hormonal findings. BTE supplementation, therefore, may not have only sped the recovery from the oxidative stress response, but may have also blunted the response as the anticipated increase in appearance of oxidative stress and inflammatory markers with increase in workload was not observed. Future research on BTE supplementation should focus on the link between the acute physiological effects and the long-term outcome of increased anaerobic exercise performance over a longer duration of time. Evaluating the effect of BTE supplementation on the performance of progressive anaerobic exercise training would aid in elucidating the pathway from reduced oxidative stress, HPA recovery, and DOMS responses to increased performance and enhanced fitness.

Inflammation, oxidative stress, and the occurrence of DOMS following high intensity anaerobic exercise are essential processes for acquisition of strength and muscle hypertrophy after exercise [1,10,12]. In excess, these responses delay recovery and result in reduced power and performance. In theory, improved training and performance would result from reducing the length of recovery and/or the extent of muscle damage after a high-intensity exercise bout.

Theaflavins, found in black tea extract, have been observed to have antioxidant effects [4] as well as anti-inflammatory effects [8,19]. Multiple epidemiological studies have found an inverse association with black tea consumption and chronic disease incidence/mortality including: congestive heart disease, stroke, atherosclerosis, pancreatic, bladder, and prostate cancers [7]. These findings have led to numerous studies examining the antioxidant effects of tea polyphenols, with emphasis on green tea and its catechins, in several models of disease.

The use of antioxidants to improve exercise performance and reduce muscle soreness is not a new concept. In this capacity, the antioxidant effects of vitamin C and E have been extensively researched [16-18]. Green tea extract (GTE) and its effects on exercise capacity and metabolism have been examined in mouse models. The duration of treadmill running was prolonged in BALB/c mice given GTE [23]. Exercise combined with GTE had a synergistic effect in attenuating high fat diet induced obesity in C57BL/6J mice [24]. GTE supplementation also increased the muscle fiber strength in a dystrophic muscle mouse model [25]. The proposed mechanisms in these studies all include the antioxidant effects of the tea polyphenols within the green tea extract. Results from recent studies have negated the common assumption that black tea has less antioxidant activity than green tea [26,27]. These previous GTE studies provide support for the ability of tea polyphenols to affect oxidative stress. Tea is one of the most widely consumed beverages in the world, and 80% of tea production results in black tea [28], designating it the most widely accepted type of tea. Our study is one of the first to examine the effects of the black tea polyphenol, theaflavin, on exercise-induced oxidative stress and inflammation in the human exercise model.

## Conclusions

The purpose of this study was to examine the effects of supplementing with a theaflavin-enriched black tea extract on DOMS, oxidative stress, inflammatory, and cortisol responses to a high intensity, anaerobic exercise protocol. The main findings in this double-blind, placebo controlled, crossover pilot study are that BTE supplementation resulted in increased performance, reduced ratings of DOMS, decreased oxidative stress markers, and improved HPA axis recovery in response to acute bouts of high-intensity exercise. This has potential application for recovery from high-intensity exercise, particularly if using repeated anaerobic intervals. Improved recovery may ultimately promote increased training frequency and quality, thus leading to improved performance.

## Competing interests

This study was funded by WellGen, Inc. (USA) through an unrestricted research grant to Rutgers, The State University of New Jersey. All researchers involved impartially collected, analyzed, and interpreted the data from this study and have no financial interests concerning the outcome of this investigation. The results from this study do not represent support by the authors and their institutions concerning the supplement investigated.

## Authors' contributions

SMA conceived of and designed this study, contributed to the acquisition, analysis and interpretation of data, led the drafting and revising of the manuscript, and gave final approval of the version to be published. MS contributed to the acquisition of data as well as the drafting and revising of the manuscript. DLG contributed to the drafting and revising of the manuscript, and gave final approval of the version to be published. KHM contributed to the design of the study and gave final approval of the version to be published.
